# Toughness Enhancement of PHBV/TPU/Cellulose Compounds with Reactive Additives for Compostable Injected Parts in Industrial Applications

**DOI:** 10.3390/ijms19072102

**Published:** 2018-07-19

**Authors:** Estefanía Lidón Sánchez-Safont, Alex Arrillaga, Jon Anakabe, Luis Cabedo, Jose Gamez-Perez

**Affiliations:** 1 Polymers and Advanced Materials Group (PIMA), Universitat Jaume I, 12071 Castellón, Spain; esafont@uji.es (E.L.S.-S.); lcabedo@uji.es (L.C.); 2 Leartiker S. Coop., Xemein Etorbidea 12A, 48270 Markina-Xemein, Spain; aarrillaga@leartiker.com (A.A.); janakabe@leartiker.com (J.A.)

**Keywords:** biopolyester, compatibilizer, cellulose, elastomer, toughening, biodisintegration, heat deflection temperature

## Abstract

Poly(3-hydroxybutyrate-co-3-valerate), PHBV, is a bacterial thermoplastic biopolyester that possesses interesting thermal and mechanical properties. As it is fully biodegradable, it could be an alternative to the use of commodities in single-use applications or in those intended for composting at their end of life. Two big drawbacks of PHBV are its low impact toughness and its high cost, which limit its potential applications. In this work, we proposed the use of a PHBV-based compound with purified α-cellulose fibres and a thermoplastic polyurethane (TPU), with the purpose of improving the performance of PHBV in terms of balanced heat resistance, stiffness, and toughness. Three reactive agents with different functionalities have been tested in these compounds: hexametylene diisocianate (HMDI), a commercial multi-epoxy-functionalized styrene-co-glycidyl methacrylate oligomer (Joncryl^®^ ADR-4368), and triglycidyl isocyanurate (TGIC). The results indicate that the reactive agents play a main role of compatibilizers among the phases of the PHBV/TPU/cellulose compounds. HMDI showed the highest ability to compatibilize the cellulose and the PHBV in the compounds, with the topmost values of deformation at break, static toughness, and impact strength. Joncryl^®^ and TGIC, on the other hand, seemed to enhance the compatibility between the fibres and the polymer matrix as well as the TPU within the PHBV.

## 1. Introduction

Nowadays, the use of plastics is widely extended in almost all production fields, such as packaging, electronics, automotive, household, etc., and the market is dominated by the so-called commodities, traditional oil-based plastics. The growing concern over the environmental problems involved with petroleum-based polymers related to their non-renewable origin and poor biodegradability is leading the industry to replace current materials with biodegradable alternatives [1]. Therefore, researchers have been looking for alternatives that may be more environmentally sustainable, especially in short- and medium-term applications, such as packaging. Within this context, biopolyesters have received great attention, especially those that are bio-sourced and biodegradable, as a way to overcome some of the waste management issues [2].

Among the different commercially available biopolyesters, one of the most promising candidates to replace commodities is the poly(3-hydroxybutyrate-co-3-valerate) (PHBV) [3,4,5,6]. PHBV is a bacterial thermoplastic biopolyester from the polyhydroxyalcanoates family that possesses physical properties comparable to conventional polyolefins, high static mechanical performance [7], and relatively high thermal resistance [8], while being fully biodegradable. However, two important drawbacks of PHBV are its low impact toughness and its cost, which is still quite high [9,10]. These disadvantages are a serious handicap for its use in applications in rigid packaging parts, for instance, that could be obtained by injection moulding. 

One of the most promising eco-friendly approaches to reduce the manufacturing costs of PHBV while maintaining its biodegradability and sustainability is the development of natural fibre-based polymer composites. Indeed, it also improves its mechanical performance in terms of stiffness as well as thermal resistance [11]. On the other hand, in order to enhance the toughness of PHBV, several attempts have been reported in the literature, some of them related to blending with other polymers such as poly(butylene adipate-co-terephthalate) (PBAT) [12,13], polybutylene succinate (PBS) [14,15], or polycaprolactone (PCL) [12] or by the addition of impact modifiers such as ethylene vinyl acetate [16], epoxidized natural rubber [13,17], or thermoplastic polyurethane (TPU) [12,18,19,20], showing in all cases great improvements in elongation at break.

In this work, we proposed the use of a purified α-cellulose fibres and a thermoplastic polyurethane (TPU) with the purpose of improving the performance of PHBV in terms of balanced heat resistance, stiffness, and toughness without compromising biodisintegrability in composting conditions. 

However, previous works have shown that the interaction of these fillers with PHA matrices was not very strong, resulting in low toughness and tensile strength [21,22]. Nonetheless, some strategies to improve the chemical affinity between the cellulose and other polyesters have been used in order to increase the reinforcement effect of the cellulose, such as fibre treatments or use of compatibilizers (reviewed by [11,23,24,25,26]).

**Figure 1:**
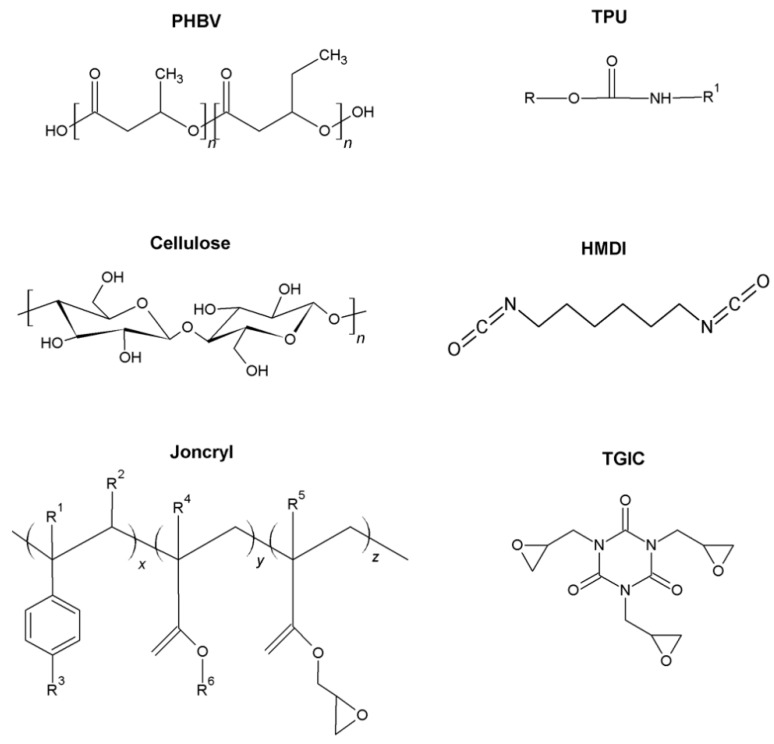
Chemical structures of materials used in this study.

From an industrial point of view, reactive extrusion is a convenient, cost-effective approach to improve the interfacial adhesion of the different phases via an in situ reaction during melt processing [27]. Within this objective, three reactive agents have been tested: (a) hexametylene diisocianate (HMDI); (b) (Joncryl^®^ ADR-4368), a commercial multi-epoxy-functionalized styrene-acrylic oligomer; and (c) triglycidyl isocyanurate (TGIC) (Figure 1). These reactive agents possess three different functional groups that could potentially react with the hydroxyl groups present at the cellulose surface and the ones from the alcohol and carboxylic acid groups at the polymer chain ends [28]. Some reports have been found in the literature about the use of diisocyanates as compatibilizers in biopolyester/fibre composites [29,30,31], PHBV/polylactic acid (PLA) blends [32], and PLA/TPU blends [33,34], showing good improvements in interfacial adhesion. Hao et al. showed improved interfacial adhesion in PLA/sisal fibre composites using Joncryl^®^ [35] and Nanthananon et al. reported similar improvements in PLA/eucalyptus fibre systems [36]. Furthermore, the use of Joncryl^®^ has also been proved efficient in the compatibilization of POM/TPU blends [37]. TGIC was successfully used to compatibilize polylactide/sisal fibre biocomposites [38].

In this work, the combined effect of TPU, cellulose fibres, and the use of three different reactive agents (HMDI, Joncryl^®^, and TGIC) is explored in order to improve the interfacial adhesion and compatibility of PHBV, TPU, and cellulose through reactive extrusion. This strategy is aimed at building a ternary system that will overcome the handicaps of PHBV that prevent its usage in injection-moulded applications in terms of cost, toughness, and thermal resistance. 

## 2. Results and Discussion

### 2.1. Preparation of Compounds and Analysis of Their Processability

PHBV/TPU/Cellulose triple systems with different content of additives (TPU and cellulose) and reactive agents (HMDI, Joncryl^®^, and TGIC) were prepared by a co-rotating twin-screw extruder in the proportions described in Table 1.

**Figure 2:**
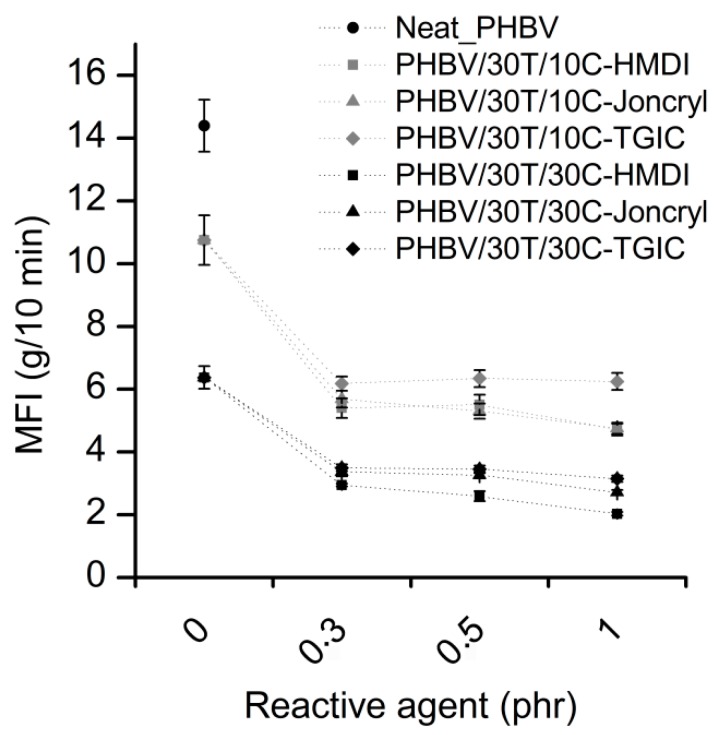
Melt flow index (MFI) of neat PHBV, PHBV/30T/10C, and PHBV/30T/30C with 0, 0.3, 0.5, and 1 phr reactive agent content.

The melt flow index is a useful tool to predict the processability of materials in industrial equipment such as injection moulding and gives an indirect measurement of melt viscosity, as it is indirectly proportional to viscosity. Figure 2 represents the melt flow index values of neat PHBV and the compounds (PHBV/30T/10C and PHBV/30T/30C) with 0, 0.3, 0.5, and 1 phr reactive agents content. 

As seen in Figure 2, the addition of TPU and cellulose significantly decreases the melt fluidity of PHBV, especially for the highest cellulose content. This increment in melt viscosity is typical in fibre-based composites because of the increased shear produced by the restricted chain mobility induced by the fibres [39]. The addition of the different reactive agents in 0.3 phr leads to a further drastic reduction of the melt fluidity. As the reactive agent content increases, the MFI values decrease slightly, except in the case of TGIC, where the compounds have similar melt indexes. Among the three reactive agents, the highest reduction in MFI is found in the compositions with HMDI. This reduction in fluidity with the incorporation of the reactive agents is indicative of some reactivity with the components of the system and can be related to a compatibilization between the fibres and the polymers and/or between the PHBV and the TPU [27].

With respect to the processability, the reduced fluidity of the compositions with the reactive agents led to increased injection pressure values. However, despite the low MFI values of the compounds, the injected samples were successfully obtained without any change in the processing parameters with respect to neat PHBV.

### 2.2. Characterization

**Figure 3:**
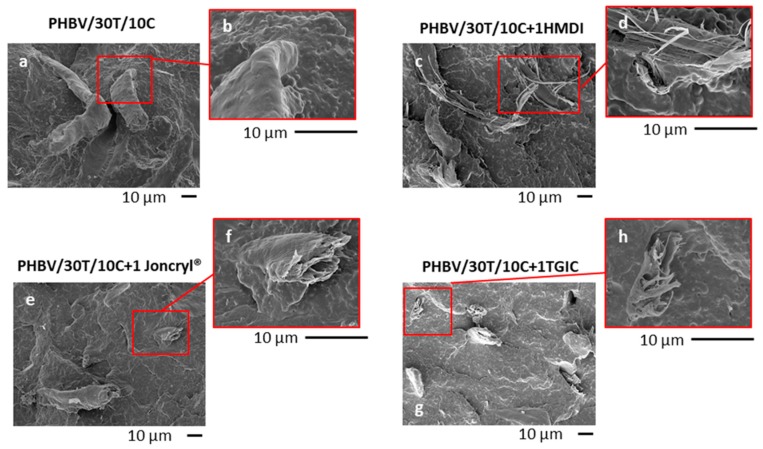
SEM micrographs of PHBV/30T/10C (**a**,**b**) and PHBV/30T/10C with 1 phr of HMDI (**c**,**d**), Joncryl^®^ (**e**,**f**), and TGIC (**g**,**h**).

**Figure 4:**
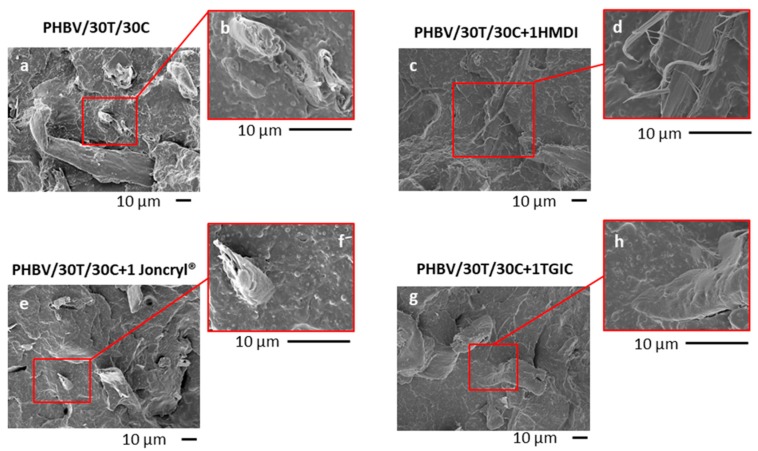
SEM micrographs of PHBV/30T/30C (**a**,**b**) and PHBV/30T/30C with 1 phr of HMDI (**c**,**d**), Joncryl^®^ (**e**,**f**), and TGIC (**g**,**h**).

The morphology of the PHBV/TPU/cellulose triple systems was analysed by scanning electron microscopy (SEM). Micrographs of PHBV/30T/10C and PHBV/30T/30C without reactive agents and with 1 phr of HMDI, Joncryl^®^, and TGIC are depicted in Figure 3 and Figure 4, respectively.

With respect to the fillers, in samples without reactive agent (Figure 3a and Figure 4a), a good distribution of the fibres was observed, indicating good compounding and certain affinity between the fibres and the polymer matrix probably due to the formation of hydrogen bonds between the –OH groups of the fibre surface and PHBV [21,40,41]. However, some detachment of the fibres is also observed, indicating that the hydrogen bonding type is not enough to provide a strong adhesion between these phases. Regarding the fibre distribution, no remarkable differences were observed with the addition of the different reactive agents (Figure 3 and Figure 4). Nevertheless, regarding the fibre–matrix interface, with the presence of the reactive agents the fibres seem to be well trapped by the polymer matrix, as broken fibres covered by the polymer were observed in all cases. In particular, in the case of HMDI (Figure 3c,d and Figure 4c,d), the fibres appear broken in the longitudinal direction, and defibrillation was observed, indicating a cohesive failure. These observations suggest the strongest adhesion between the cellulose fibres and the PHBV, indicating a compatibilization effect.

**Figure 5:**
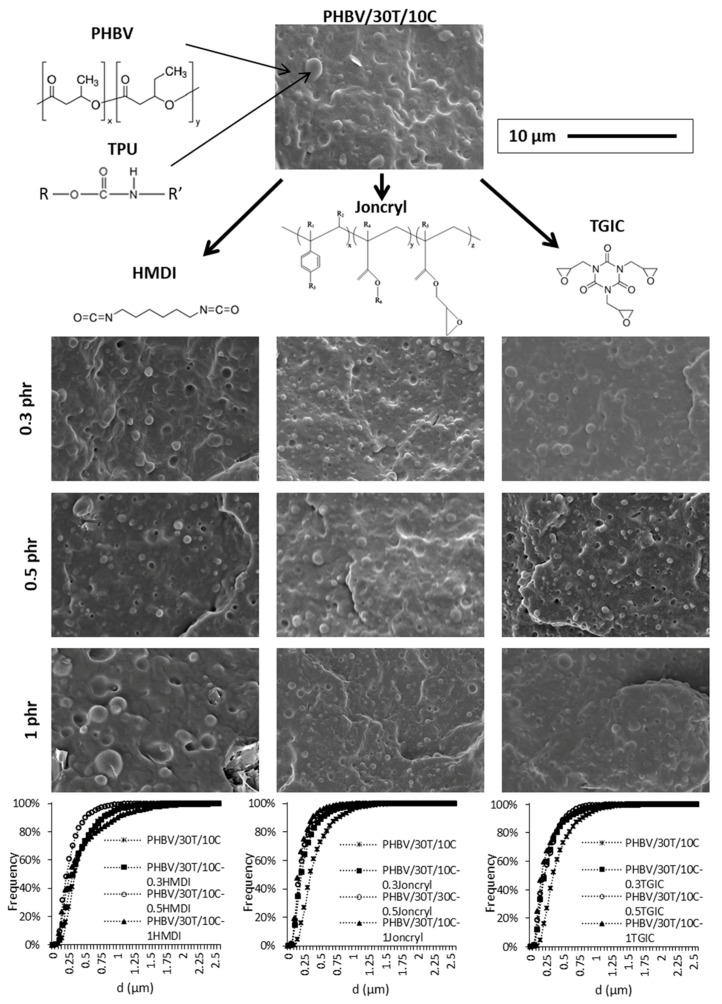
SEM images of the PHBV/30T/10C composites with the different reactive agents and cumulative frequency droplet size histograms of the dispersed phase.

**Figure 6:**
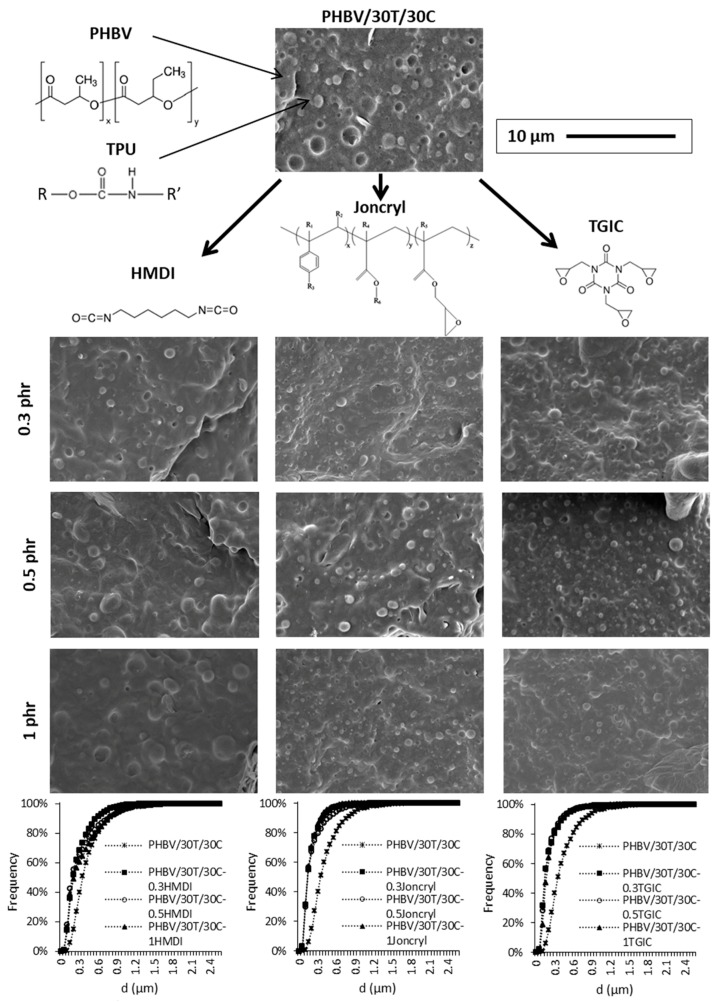
SEM images of the PHBV/30T/30C composites with the different reactive agents and cumulative frequency droplet size histograms of the dispersed phase.

With respect to the polymeric matrix, all the compositions present a drop in matrix morphology, where the disperse phase is the TPU, as shown in Figure 5 and Figure 6. These figures show the SEM images of the polymeric matrix for the PHBV/30T/10 and PHBV/30T/30C composites, with and without the reactive agents. The droplet size distributions of the dispersed phase are also included in the aforementioned figures. The average domain size (*d*), the estimated ligament distance, and the d10, d50, and d90 values are summarized in Table 2.

According to the measurements performed, the average domain size (*d*) of the TPU is 0.416 and 0.420 μm in PHBV/30T/10C and PHBV/30T/30C, respectively. Although some detachment of TPU is observed, the small size of the dispersed phase domains indicates a certain affinity between the phases. With the incorporation of the reactive agents the average TPU droplet size was reduced, as shown in Table 2. The highest droplet size reductions were obtained in the compounds containing the highest amount of cellulose with the reactive agents TGIC and Joncryl^®^. In these cases, indeed, a slight dependence on the average domain size (*d*) as the reactive agent content increases was observed in compounds with 10 phr of Cellullose, but not in those with 30 phr Cellulose.

Regarding compounds with HMDI, the reduction in the average domain size (*d*) is lower with respect to the other reactive agents. In fact, the compound PHBV/30T/10C with 1 phr HMDI shows a similar value of *d* as the compound without reactive agents, with the domain size distribution being slightly displaced to bigger sizes, presenting the highest d90 value among all compounds. Nevertheless, the matrix ligament thickness of this system is in the same range as in the rest of the composites.

Despite the differences in the size distributions, the droplet size and the estimated matrix ligament thickness (T) are quite small for all compositions. As reported by Wu [42], the matrix ligament thickness plays an important role for rubber toughening in polymer blends. If the average matrix ligament thickness, defined as the surface-to-surface interparticle distance, is smaller than a critical value, the blend will be tough, whereas on the contrary the blend will be brittle. It can be concluded, from this analysis, that the use of TGIC and Joncryl^®^ reactive agents produced an enhanced compatibilization effect on the TPU domains within the PHBV/cellulose matrix.

#### 2.2.1. Mechanical Properties

**Figure 7:**
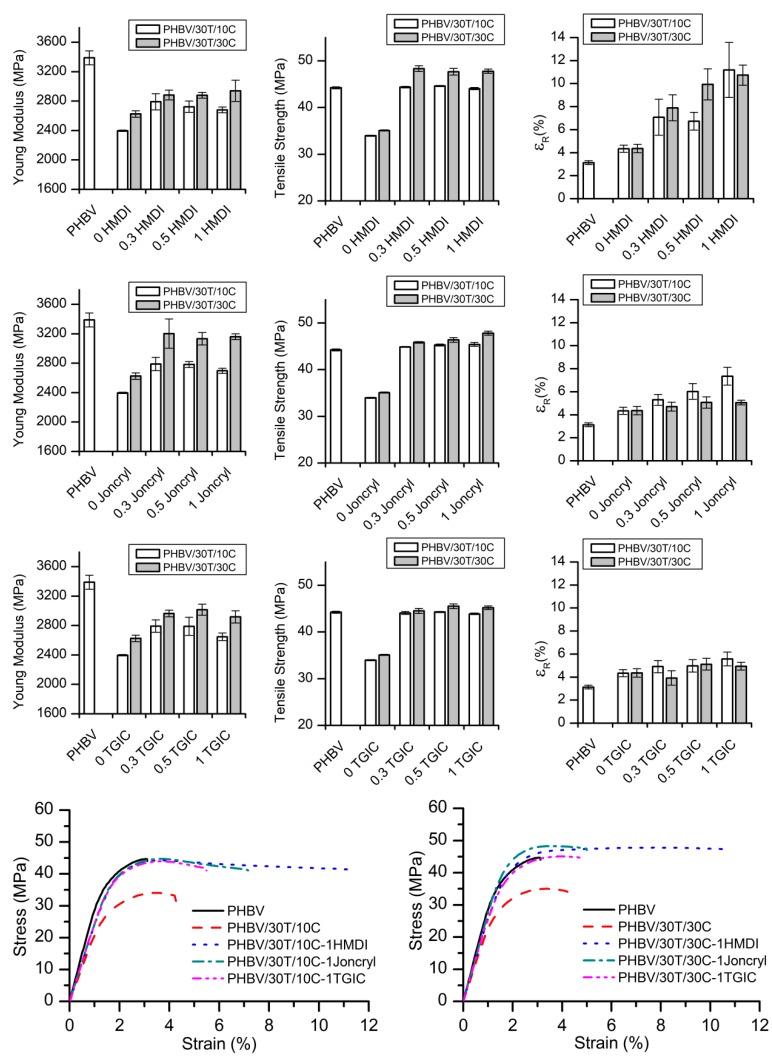
Mechanical properties of the neat PHBV and PHBV/TPU/Cellulose blends and representative stress–strain curves of neat PHBV, PHBV/30T/10C, and PHBV/30T/30C systems with and without 1 phr reactive agents.

Tensile tests up to failure were conducted in order to study the mechanical properties of neat PHBV and the compounds with and without reactive agents (HMDI, Joncryl^®^, and TGIC). The Young’s modulus, tensile yield strength, and elongation at break of the different compositions are shown in Figure 7. The representative strain–stress curves of neat PHBV and PHBV/TPU/cellulose composites with and without the highest level of reactive agents (1 phr) are also represented for the sake of clarity.

PHBV presents a typical stiff and brittle mechanical performance, with high values of elastic modulus and tensile strength and low elongation at break (<5%). With respect to neat PHBV, the incorporation of TPU (30 phr) and cellulose (10 or 30 phr) leads to a reduction in the rigidity (about 25%) and the tensile strength (about 20%) and an enhancement in elongation at break (ca. 40%) and static toughness (25% and 32%, respectively) [43]. This increase in elongation at break is related with both the good distribution and small droplet size of the dispersed elastomeric phase (TPU) [19,20]. On the other hand, although there is a certain affinity among the phases, this limited interaction is not enough to ensure an efficient load transfer to the cellulose fibres. Without strong adhesion, the fibres detach at low deformation values, lowering the tensile strength of the compound and acting as stress concentrators for premature material failure [44]. With the addition of the reactive agents to the compounds, the elastic modulus and the tensile strength clearly increase with respect to the PHBV/TPU/cellulose without them. These parameters are strongly influenced by the matrix–fibre interaction and their improvement indicates a better load transfer due to an enhanced adhesion [30]. For the 10 phr cellulose compounds, the different reactive agents show a similar impact in these parameters, regardless of their content, supposing an improvement in the elastic modulus of about 15% and an increase in the tensile strength of around 30%. For the 30 phr cellulose compounds, the highest rise in the tensile modulus was obtained with the addition of Joncryl^®^ (20% vs. ca. 10% for HMDI and TGIC). On the other hand, the tensile strength was improved by around 40%, 30%, and 35% with the HMDI, Joncryl^®^, and TGIC, respectively, reaching that of neat PHBV.

These results reveal that the three tested reactive agents are effective at improving the interfacial adhesion of the cellulose with the polymeric matrix and are in accordance with the SEM observations and the MFI values, pointing to an increased interaction among the phases [45]. This conclusion is in agreement with some other works that have been reported in the literature, on biopolyester–fibre composites compatibilized with diisocyanates [29,30,31], Joncryl^®^ [35,36], or TGIC [38].

The biggest difference among the tested reactive agents with respect to their influence on the mechanical performance of the compounds is in the elongation at break. In all cases, this parameter was improved with respect to both neat PHBV and the compound without reactive agents. In Figure 6, looking at any PHBV/30T/10C compounds, as the reactive agent addition increases, the elongation at break rises too. On the other hand, in the case of the PHBV/30T/30C compounds, only the compounds with HMDI show an increase of elongation at break as the reactive agent content increases. This difference may point that the role of HMDI may not be the same as TGIC or Joncryl^®^.

In fact, the compounds with the highest TGIC level (1 phr) show an increase in elongation at break of ca. 28% with respect to the uncompatibilized PHBV/30T/10C system and 13% for PHBV/30T/30C. Similarly, the addition of 1 phr Joncryl^®^ improved elongation at break by 70% in PHBV/30T/10C and 16% in PHBV/30T/30C. However, the compounds with 1 phr of HMDI showed an extraordinary enhancement of the elongation at break; the elongation at break was improved by 160% and 150% for compounds with 10 and 30 phr cellulose, respectively. Moreover, the static toughness (calculated from the area below the stress–strain curve) was enhanced by 320% and 340% with respect to the compound without reactive agents, and 420% and 450% with respect to neat PHBV.

#### 2.2.2. Impact Resistance

**Figure 8:**
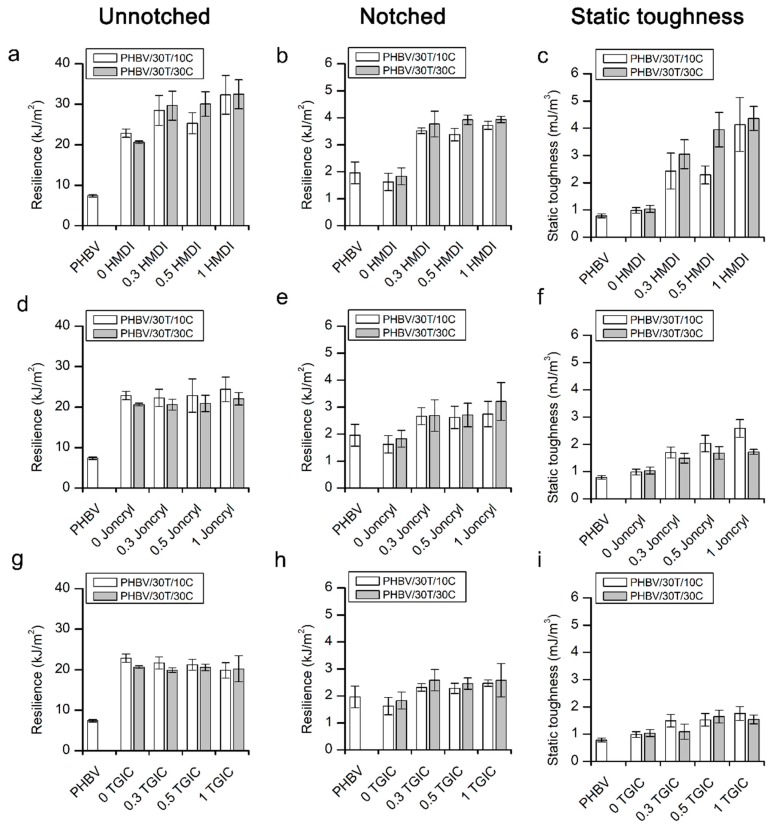
Charpy’s impact results for unnotched specimens (**a**,**d**,**g**), notched specimens (**b**,**e**,**h**) and static toughness from the area below the strain–stress curve (**c**,**f**,**i**).

Figure 8 summarizes the values obtained from unnotched and notched Charpy’s impact tests, along with the static toughness from tensile tests of neat PHBV and the compounds with and without the reactive agents.

It is known that PHBV is very brittle and therefore it presents very low values of resilience in both unnotched and notched Charpy’s impact tests and low static toughness, as shown in Figure 7. In this figure it can be observed that the compounds with TPU and cellulose clearly show an improvement in toughness resistance in the case of unnotched impact tests (Figure 8a,d,g) [43]. This improvement could be attributed to the positive role that the elastomeric TPU phase plays in absorbing impact energy [46]. However, concerning the notched tests (Figure 8b,e,h), there is no such increase in the impact energy absorbed, probably because there is a preferred crack propagation pathway through the matrix/fibre interfaces, where the adhesion is not very strong, as previously pointed out when discussing the variations in the elastic modulus of the compounds.

Nevertheless, the addition of reactive agents significantly improves the notched impact properties of the composites, which is in agreement with the static toughness determined from the area below the stress–strain curves (Figure 8c,f,i). This enhancement of the matrix–fibre interface adds to the effect of the small droplet size of the elastomeric phase that implies a low ligament thickness [42,47] and to by the enhanced interfacial interactions between TPU and PHBV [37], resulting in higher impact resistance in the presence of a notch.

When analysing the influence of the different reactive agents, the greatest increase in impact strength is obtained for composites with HMDI. For these composites, the absorbed impact energy was highly improved in both unnotched and notched impact tests, as well as in the static toughness (Figure 8a–c). Attending to the SEM micrographs of the impact fractured surfaces, with the HMDI addition (Figure 3 and Figure 4) most of the fibres appear broken at their longitudinal direction, thus indicating a cohesive failure that confirms the presence of a very strong interface. This was not the case in compounds with the addition of TGIC or Joncryl^®^.

In polymer matrix composites, when there is a weak interface between the second phase and the polymeric matrix, the detachment of the particles during tensile deformations leads to the formation of flaws and voids at the interface of the fibre and the matrix. Those voids can coalesce and act as either crack initiators or provide a fast propagation crack pathway, which eventually leads to the premature failure of the material [13,48]. With a stronger particle–matrix adhesion, the possibility of growth and merge of those internal flaws is reduced, so there is an effective load transfer between the two phases, improving the fracture toughness [30]. It can be said that when the shear strength at the particle–matrix interface is higher than the shear yielding of any of the phases, plastic deformation of any of them can occur, thus increasing the energy absorbed. Thus, reactive agents can play different roles, increasing the adhesion between the PHBV/TPU, PHBV/cellulose and TPU/cellulose interfaces.

The impact performance of the compounds with HMDI stands out over the other ones. In this case, it seems that HMDI strongly increases the adhesion between PHBV and cellulose, which results in a synergetic effect with the addition of the TPU. The well-dispersed elastomeric phase decreases the yield strength of the polymer matrix and the strong interaction between the polymer and the fibres allows effective load transfer without producing flaws at the interfaces. Moreover, the exceptional mechanical performance of these compositions in terms of elongation at break also suggests that HMDI could play a positive role in enhancing the interfacial adhesion between PHBV and TPU. Indeed, diisocyanates have demonstrated effectiveness in improving the compatibility of biopolyester/TPU blends, as has been reported by Dogan et al. [33,34].

TGIC and Joncryl^®^, according to this reasoning, would not be so effective at enhancing the cellulose/PHBV interface, thus showing limited values of impact resistance, especially in the presence of a notch.

#### 2.2.3. Heat Deflection Temperature HDT-A

The thermal resistance of neat PHBV and PHBV/TPU/cellulose composites was evaluated by means of heat deflection temperature (HDT-A) measurements. The results are grouped in Table 3.

The PHBV presents a relatively high thermal resistance, showing an HDT-A value of 108 °C, in agreement with previously reported values [8,49,50]. The HDT values obtained for PHBV/30T/10C and PHBV/30T/30C are 94 and 96 °C, respectively. In spite of the relatively high content of the elastomeric additive (30 phr), the thermal resistance is not that much lower. This is due to the positive role played by the cellulose fibres in terms of reinforcement. As is widely reported in literature, in fibre-based polymer composites the restricted mobility of polymer chains in the presence of fibres leads to an increase in the dimensional stability and, thus, higher temperatures are required to deform them [50].

The use of reactive agents did have a significant influence on HDT values, but a trend was not seen with variation on their relative content. For the compounds with the lowest cellulose content (10 phr), HDT values ranged between 90 °C (TGIC) and 98 °C (Joncryl^®^), compared with a value of 94 °C for the compound without reactive agents. On the other hand, for the PHBV/30T/30C compounds, the thermal resistance was in almost all cases improved with HDT-A values around 100 °C (especially HMDI and TGIC), being the HDT value for the compound without reactive agents 96 °C.

These results are in agreement with the improved rigidity of the samples in the presence of reactive agents. Indeed, since there is no dependence of the HDT value on increasing the content of reactive agents, crosslinking reactions among the polymer chains can be discarded, thus indicating that the effect of the reactive agents is only a consequence of the compatibilization of the different phases. The increase in the HDT of fibre-based composites is therefore related to the reinforcement of the cellulose fibres [51] and, along with the use of the studied reactive agents, allows for enhancing the toughness and mechanical performance of PHBV without drastically decreasing its thermal resistance. 

#### 2.2.4. Biodisintegration in Composting Conditions

**Figure 9:**
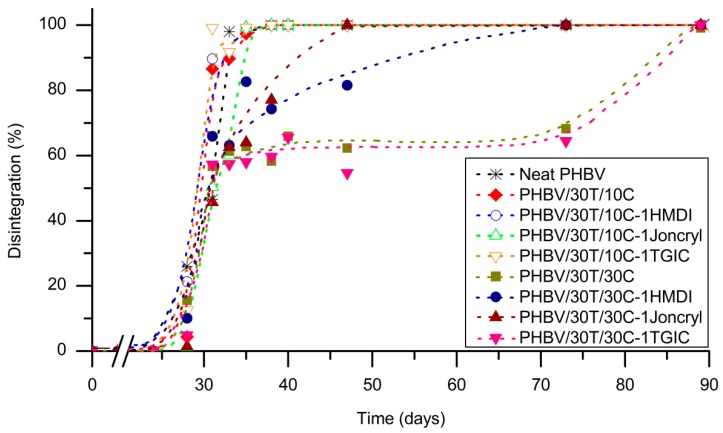
Disintegration in composting conditions of neat PHBV and PHB/TPU/cellulose systems with and without 1 phr reactive agents.

To explore the influence of cellulose content and the different reactive agents on the compostability of the PHBV/TPU/cellulose ternary systems, biodisintegration tests were conducted according to the ISO 20200 standard. The disintegration (weight loss) level over composting time is represented in Figure 9.

In general, all the compositions studied can be considered biodisintegrable in composting conditions according to ISO 20200. As shown in Figure 9, the PHBV disintegration process starts after an incubation period of 28 days. At this time the disintegration rate drastically increases to achieve total disintegration at 38 days of composting, in accordance with previous works [20,52,53]. No differences in the biodisintegration rate were detected for the PHBV/TPU/cellulose composites containing 10 phr cellulose, independent of the presence of reactive agents or the reactive agent type.

When the cellulose content was increased, the biodisintegration rate was, oddly, significantly reduced. To understand this occurrence, it must be taken into account how the samples were prepared and how biodisintegration takes place. For the composting tests, the specimens were obtained by hot pressing. Under the hot pressing conditions, the formation of a percolation mesh of interconnected cellulose fibres is favoured due to the high fibre–fibre affinity of the cellulose. This cellulose mesh is partially covered by TPU, which possess a low biodisintegration rate [20] with respect to PHBV and cellulose. Then, during the incubation time, a biofilm is formed at the surface of the testing specimen and the microbial advance occurs from the surface to the bulk, preferentially through the PHBV phase, as it is deduced by the stabilization of the disintegrated mass at around 60 wt % (approximately, the PHBV weight content) after 35 days of composting. We think that the TPU droplets, which take longer to biodisintegrate and are quite sticky at high temperature and moisture content, cover the fibres, limiting the access of the microbial advance to the cellulose.

**Figure 10:**
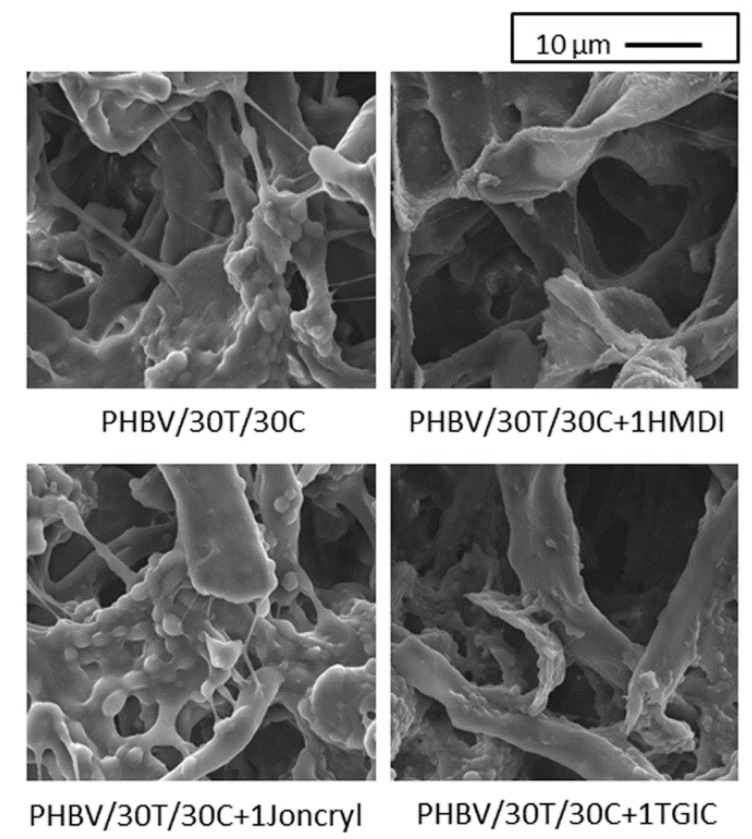
SEM micrographs of PHBV/30T/30C samples with and without 1 phr reactive agents after Soxhlet extraction.

This phenomenon causes a slowdown in the biodisintegration rate, but when the microorganisms have access to cellulose the weight loss rises rapidly and total disintegration is achieved within 90 days of composting. To validate this hypothesis, similar samples to those used for biodisintegration were placed for Soxhlet extraction of the PHBV phase with chloroform, and the resulting morphology analysed by SEM (Figure 10). TPU can be seen covering the fibres, partially confirming this reasoning.

Furthermore, PHBV/30T/30C composites showed different behaviour depending on the reactive agent added. The composition with TGIC presented a similar trend to the composition without reactive agent (that is, a slowdown at 60% weight loss); the ones with HMDI presented a fast biodisintegration of about 80% after 33 days of composting, reaching complete disintegration at day 73; and the composition with Joncryl^®^ was totally degraded after 47 days. These differences in the biodisintegration rates among PHBV/30T/30C composites with different reactive agents could be influenced by the interactions of the reactive agents with the fibres, the PHBV, and the TPU. It is hypothesized that when there is a high interaction between the cellulose and the PHBV (promoted by the reactive agents), the microorganisms can access the cellulose more easily and the biodisintegration is completed earlier. On the contrary, when the PHBV–cellulose interaction is weak, the TPU can be easily located at the fibres surface, hindering the microbial advance. When looking at the SEM pictures of the compounds after Soxhlet extraction (Figure 10), there is more polymer covering the fibres in the case of no reactive agent or TGIC addition than in the case of the compounds with HMDI and Joncryl^®^, supporting the aforementioned reasoning.

## 3. Experimental

### 3.1. Materials

Poly(3-hydroxybutyrate-co-3-hydroxyvalerate) (PHBV) commercial grade with 3 wt % valerate content was purchased from Tianan Biologic Material Co. (Ningbo, China) in pellet form (ENMAT Y1000P). Thermoplastic polyurethane (TPU) Elastollan^®^ 890 A 10FC was supplied by BASF (Ludwigshafen, Germany). Purified alpha-cellulose fibre grade (TC90) (alpha-cellulose content >99.5%) from CreaFill Fibers Corp. (Chestertown, MD, USA) was used. The reactive agents hexamethylene diisocyanate (HMDI) and triglycidyl isocyanurate (TGIC) were supplied by Sigma-Aldrich (Spain) and the Joncryl^®^ 4368 was purchased from BASF (Ludwigshafen, Germany).

### 3.2. Sample Preparation

The PHBV and TPU used in this study were dried at 80 °C for at least 6 h in a DESTA DS06 HT dehumidifying dryer and the cellulose was dried in a lab oven (Memmert universal oven U, Schwabach, Germany) at 90 °C for a minimum of 16 h prior to the blending step, whilst the three reactive agents (HMDI, Joncryl^®^, and TGIC) were used as received. 

PHBV/TPU/cellulose triple systems with different content of additives (TPU and cellulose) and reactive agents (HMDI, Joncryl^®^, and TGIC) (see Table 1) were prepared in a Labtech LTE (Samutprakarn, Thailand) (Ø = 26 mm, L/D ratio = 40) co-rotating twin-screw extruder. The temperature profile was set at 145/155/160/170 °C from hopper to nozzle, the rotation speed was 250 rpm, and the feeding speed was about 5 kg/h. All the components were manually dry-mixed before extrusion except HMDI, which was dispensed at the feeding zone by means of a peristaltic pump (Watson Marlow 120 S/R, Sondika, Spain). The extruded material was cooled in a water bath and pelletized (MAAG PRIMO S pelletizer, Stuttgart, Germany).

Material pellets were dried again at 80 °C for 8 h (DESTA DS06 HT) before the injection process. Standardized tensile specimens (ISO-527 Type 1A) were injection-moulded in a DEMAG IntElect 100 T injection moulding machine (Schwaig, Germany) with an injection temperature of 185 °C at the nozzle. A holding pressure of 600 bars was applied for 12 s, followed by 40 s of cooling time. For the sake of comparison, neat PHBV was also processed under identical conditions.

Prior to any characterization, all the samples were annealed at 80 °C for 48 h in order to obtain equivalent crystallinity and mechanical performance to aged samples.

### 3.3. Characterization

The melt flow index (MFI) of the different compounds was measured in a Tinius Olsen MP600 (Surrey, England) melt flow indexer according to the ISO 1133 standard. The tests were performed at 185 °C and 2.16 kg load.

The morphology of the PHBV/30T/10C and PHBV/30T/30C triple systems with and without reactive agents (HMDI, Joncryl^®^ and TGIC) was examined by scanning electron microscopy (SEM) using a high-resolution field-emission JEOL 7001F microscope (Japan). The fracture surfaces from impact-fractured specimens were previously coated by sputtering with a thin layer of Pt. From selected representative SEM images (at 2500× magnification), the diameters of the droplets corresponding to the dispersed phase were measured using Fiji^®^ software (ImageJ 1.51j8). The number of droplets measured in all cases was higher than 600. From the individual measures, the following parameters were determined: the average droplet size (*d*) and the droplet size distribution parameters d10, d50, and d90 (corresponding to the size where 10%, 50%, and 90% of the droplets are included, respectively). The matrix ligament thickness (*T*) was also estimated, according to Wu’s equation [42]:

(1) $$T=d\left[{\left(\frac{\pi }{6\varphi_{r}}\right)}^{\frac{1}{3}}-1\right],$$

where *d* is the average domain size of the dispersed phase and $\varphi_{r}$ is the volume fraction of the dispersed phase, determined as follows:

(2) $$\varphi_{r}=\frac{\rho_{m\;w_{r}}}{\left(\rho_{r}w_{m}+\rho_{m}w_{r}\right)},$$

where *ρ_m_* and *ρ_r_* are the densities of the matrix and dispersed phases, respectively, and *w_m_* and *w_r_* are their weight fractions. 

Tensile tests were conducted on ISO-527 type 1A injection-moulded specimens in a Hounsfield H25K universal testing machine (Surrey, England) equipped with a 25 kN load cell according to the ISO-527-1:2012 standard. 

Notched and unnotched Charpy impact tests were carried out by means of an ATS faar IMPats-15 (Segrate, Italy) impact pendulum with a 4 J hammer according to the ISO 179 standard. Samples were cut from injection-moulded bars.

Heat deflection temperature (HDT) analyses were performed using a Deflex 687-2 (Barcelona, Spain). A heating rate of 120 °C/h was used with an applied load of 1.8 MPa in accordance with Method A of ISO 75 standard. The temperature was recorded until the sample deflects 0.35 mm. 

Biodisintegration tests were carried out with samples (15 × 15 × 0.2 mm^3^) obtained from hot-pressed plates (180 °C, 5 min, and ca. 40 bar). Tests were performed according to the ISO 20200 standard [54]. Solid synthetic waste was prepared by mixing 10% of activated mature compost (VIGORHUMUS H-00, purchased from Buras Profesional, S.A., Girona, Spain), 40% sawdust, 30% rabbit feed, 10% corn starch, 5% sugar, 4% corn seed oil, and 1% urea. The water content of the mixture was adjusted to 55%. The samples were placed inside mesh bags to simplify their extraction and allow the contact of the compost with the specimens, and then were buried in compost bioreactors at 4–6 cm depth. Bioreactors were incubated at 58 °C. The aerobic conditions were guaranteed by periodically mixing the synthetic waste and adding water according to the standard requirements. Three replicates of each sample were removed from the boxes at different composting times for analysis. Samples were washed with water and dried under a vacuum at 40 °C until a constant mass. The disintegration degree was calculated by normalizing the sample weight to the initial weight with Equation (3):

(3) $$D=\frac{m_{i}-m_{f}}{m_{i}}\times 100,$$

where *m_i_* is the initial dry mass of the test material and *m_f_* is the dry mass of the test material recovered at different incubation stages. Moreover, the morphology of the films prepared for the composting tests was analysed by SEM after the Soxhlet extraction with chloroform of the PHBV phase.

## 4. Conclusions

In this study three reactive agents used in reactive extrusion (HMDI, Joncryl^®^, and TGIC) were tested in PHBV/TPU/cellulose for injection moulding applications that require biodisintegration in composting conditions. The influence of the cellulose content, the reactive agent type and the reactive agent content, were analysed. It was observed that the incorporation of TPU and cellulose in PHBV led to a reduction in the tensile elastic modulus and tensile strength, but an enhancement in elongation at break, with an overall increase in static toughness attributed to the toughening effect of the TPU. However, the addition of the reactive agents to the compounds resulted in a rise in the tensile strength and elastic modulus up to values close to or higher than neat PHBV and an increase in the value of strain at break with respect to the compounds without reactive agents.

In terms of impact resistance, the addition of the reactive agents improved the toughness of the compounds in notched and unnotched configurations. Furthermore, even though the TPU in the compounds causes a decrease in the thermal strength with respect to neat PHBV, the addition of cellulose up to 30 phr with the reactive agents was able to moderate this drop. 

Those results indicate that the reactive agents play a main role as compatibilizers among the phases of the PHBV/TPU/cellulose compounds. HMDI showed the highest ability to compatibilize the cellulose and the PHBV in the compounds, with the topmost values of deformation at break and static toughness. Joncryl^®^ and TGIC, on the other hand, seemed to enhance the compatibility between the fibres and the polymer matrix as well as the TPU within the PHBV. 

The findings of this work point to a route to modify the properties of PHBV (and PHAs in general) through blending with reactive agents, which can help to overcome some of the difficulties that these materials encounter in standard applications.

## Figures and Tables

**Table 1:** List of compounds and their composition.

Sample	TPU	Cellulose	HMDI	Joncryl^®^	TGIC
	(phr) *				
Neat PHBV	-	-	-	-	-
PHBV/30T/10C **	30	10	-	-	-
PHBV/30T/10C-0.3HMDI	30	10	0.3	-	-
PHBV/30T/10C-0.5HMDI	30	10	0.5	-	-
PHBV/30T/10C-1HMDI	30	10	1	-	-
PHBV/30T/10C-0.3Joncryl	30	10	-	0.3	-
PHBV/30T/10C-0.5Joncryl	30	10	-	0.5	-
PHBV/30T/10C-1Joncryl	30	10	-	1	-
PHBV/30T/10C-0.3TGIC	30	10	-	-	0.3
PHBV/30T/10C-0.5TGIC	30	10	-	-	0.5
PHBV/30T/10C-1TGIC	30	10	-	-	1
PHBV/30T/30C ***	30	30	-	-	-
PHBV/30T/30C-0.3HMDI	30	30	0.3	-	-
PHBV/30T/30C-0.5HMDI	30	30	0.5	-	-
PHBV/30T/30C-1HMDI	30	30	1	-	-
PHBV/30T/30C-0.3Joncryl	30	30	-	0.3	-
PHBV/30T/30C-0.5Joncryl	30	30	-	0.5	-
PHBV/30T/30C-1Joncryl	30	30	-	1	-
PHBV/30T/30C-0.3TGIC	30	30	-	-	0.3
PHBV/30T/30C-0.5TGIC	30	30	-	-	0.5
PHBV/30T/30C-1TGIC	30	30	-	-	1

* phr refers to the 100 unit weight PHBV matrix; ** PHBV/30T/10C corresponds to 71.4 wt % PHBV, 21.4 wt % TPU, and 7.2 wt % cellulose; *** PHBV/30T/30C corresponds to 62.5 wt % PHBV, 18.8 wt % TPU, and 18.8 wt % cellulose.

**Table 2:** Estimated d10, d50 and d90, average droplet size and ligament distance values of the TPU dispersed phase.

(phr)	PHBV/30T/10C					PHBV/30T/30C				
	d10 (μm)	d50 (μm)	d90 (μm)	*d* (μm)	T (μm)	d10 (μm)	d50 (μm)	d90 (μm)	*d* (μm)	T (μm)
0	0.17	0.34	0.79	0.42	0.13	0.17	0.35	0.77	0.42	0.13
0.3 HMDI	0.14	0.31	0.79	0.40	0.12	0.09	0.20	0.57	0.27	0.08
0.5 HMDI	0.11	0.21	0.51	0.26	0.08	0.09	0.18	0.76	0.31	0.10
1 HMDI	0.11	0.26	1.01	0.43	0.13	0.09	0.21	0.77	0.33	0.10
0.3 Joncryl	0.09	0.20	0.52	0.26	0.08	0.07	0.14	0.38	0.19	0.06
0.5 Joncryl	0.09	0.17	0.43	0.22	0.07	0.08	0.14	0.48	0.21	0.07
1 Joncryl	0.09	0.16	0.39	0.20	0.06	0.07	0.14	0.36	0.18	0.06
0.3 TGIC	0.10	0.21	0.57	0.28	0.09	0.07	0.14	0.43	0.20	0.06
0.5 TGIC	0.09	0.21	0.52	0.26	0.08	0.08	0.14	0.39	0.20	0.06
1 TGIC	0.08	0.17	0.54	0.25	0.08	0.09	0.17	0.42	0.21	0.07

**Table 3:** HDT-A values for neat PHBV and PHBV/TPU/cellulose systems with and without reactive agents.

Sample	HDT-A (°C)			
PHBV/TPU/Cellulose		0.3 phr	0.5phr	1 phr
PHBV	108 ± 1			
PHBV/30T/10C	94 ± 3			
PHBV/30T/10C + HMDI		93 ± 1	98 ± 3	95 ± 4
PHBV/30T/10C + Joncryl		95 ± 1	98 ± 3	97 ± 1
PHBV/30T/10C + TGIC		90 ± 3	90 ± 1	90 ± 3
PHBV/30T/30C	96 ± 1			
PHBV/30T/30C + HMDI		97 ± 2	99 ± 2	100 ± 1
PHBV/30T/30C + Joncryl		98 ± 4	97 ± 3	94 ± 1
PHBV/30T/30C + TGIC		104 ± 3	99 ± 3	99 ± 1
